# Genome-Wide Characterization and Expression Profiling of Phytosulfokine Receptor Genes (*PSKR*s) in *Triticum aestivum* with Docking Simulations of Their Interactions with Phytosulfokine (PSK): A Bioinformatics Study

**DOI:** 10.3390/genes15101306

**Published:** 2024-10-09

**Authors:** Hala Badr Khalil

**Affiliations:** 1 Department of Biological Sciences, College of Science, King Faisal University, P.O. Box 380, Al-Ahsa 31982, Saudi Arabia; hkhalil@kfu.edu.sa; 2 Department of Genetics, Faculty of Agriculture, Ain Shams University, 68 Hadayek Shoubra, Cairo 11241, Egypt

**Keywords:** docking simulation, gene expression, phytosulfokine, phytosulfokine receptors, plant development, *Triticum aestivum*

## Abstract

**Background/Objectives:** The phytosulfokine receptor (*PSKR*) gene family plays a crucial role in regulating plant growth, development, and stress response. Here, the *PSKR* gene family was characterized in *Triticum aestivum* L. The study aimed to bridge knowledge gaps and clarify the functional roles of *TaPSKRs* to create a solid foundation for examining the structure, functions, and regulatory aspects. **Methods:** The investigation involved genome-wide identification of *PSKRs* through collection and chromosomal assignment, followed by phylogenetic analysis and gene expression profiling. Additionally, interactions with their interactors were stimulated and analyzed to elucidate their function. **Results:** The wide-genome inspection of all *TaPSKRs* led to 25 genes with various homeologs, resulting in 57 *TaPSKR* members distributed among the A, B, and D subgenomes. Investigating the expression of 61 *Ta*PSKR cDNAs in RNA-seq datasets generated from different growth stages at 14, 21, and 60 days old and diverse tissues such as leaves, shoots, and roots provided further insight into their functional purposes. The expression profile of the *TaPSKRs* resulted in three key clusters. Gene cluster 1 (GC 1) is partially associated with root growth, suggesting that specific *TaPSKR*s control root development. The GC 2 cluster targeted genes that show high levels of expression in all tested leaf growth stages and the early developmental stage of the shoots and roots. Furthermore, the GC 3 cluster was composed of genes that are constantly expressed, highlighting their crucial role in regulating various processes during the entire life cycle of wheat. Molecular docking simulations showed that phytosulfokine type α (PSK-α) interacted with all *Ta*PSKRs and had a strong binding affinity with certain *Ta*PSKR proteins, encompassing *Ta*PSKR1A, *Ta*PSKR3B, and *Ta*PSKR13A, that support their involvement in PSK signaling pathways. The crucial arbitration of the affinity may depend on interactions between wheat PSK-α and PSKRs, especially in the LRR domain region. **Conclusions:** These discoveries deepened our knowledge of the role of the *TaPSKR* gene family in wheat growth and development, opening up possibilities for further studies to enhance wheat durability and yield via focused innovation approaches.

## 1. Introduction

Wheat stands as a cornerstone of human nutrition and civilization, boasting a rich history and multifaceted importance. Beyond its nutritional value, wheat’s adaptability to diverse climates and its cultural and economic significance have shaped societies for millennia. As a key player in the agricultural landscape, wheat contributes to food security and economic prosperity [1]. In the realm of agricultural innovation, harnessing the power of extraordinary biomolecules presents a promising avenue for enhancing wheat resilience. Researchers are increasingly exploring advanced molecular technologies to develop novel solutions that fortify wheat against various stressors, including pests, diseases, and environmental challenges [2]. These extraordinary biomolecules might include genetically engineered peptides, enzymes, or specialized compounds that can bolster the plant’s natural defense mechanisms [2]. By understanding the intricate molecular pathways within wheat, researchers can pinpoint specific targets for enhancement, leading to the creation of genetically modified varieties with enhanced resistance [3]. The utilization of extraordinary biomolecules not only holds the potential to revolutionize wheat agriculture but also underscores the importance of cutting-edge biotechnology in addressing the evolving challenges faced by global food systems [4].

Signal transduction plays a crucial role in plant development, growth regulation, and stress response. This intricate process is governed by various factors, including growth regulators and their associated signal transduction pathways [5]. Notably, peptide signaling in plants has emerged as a key element of cell-to-cell communication, orchestrating the coordination of growth and developmental processes. The phytosulfokine family (PSK) is a fascinating pentapeptide hormone found in plants that holds a pivotal role in orchestrating various aspects of their growth, development, and environmental challenges. Comprising just five amino acids, this molecule is synthesized from a precursor protein and acts as a potent signaling molecule between plant cells [6,7]. When one cell secretes PSK, neighboring cells perceive it and respond accordingly. One of its fundamental functions is the promotion of cell division and expansion, a crucial process in plant growth. PSK facilitates the formation of new cells and tissues, helping plants develop and adapt to their environment [8]. Within the PSK family, two primary types, PSK-α and PSK-β, have been identified [9]. PSK-α, characterized by its conserved pentapeptide sequence (Tyr (SO3H)-Ile-Tyr (SO3H)-Thr-Gln), is recognized for its multifaceted functions [6]. It is involved in cell division and expansion, influencing processes such as root development and lateral root growth [10]. Furthermore, PSK-α plays a crucial role in modulating plant immune responses, contributing to defense mechanisms against various pathogens [9,11]. The PSK-α peptide is an intriguing molecular entity derived from the cleavage of preproproteins composed of approximately 80 amino acids. This process yields mature PSK, a pentapeptide consisting of precisely five amino acids, notably featuring two sulfated tyrosine residues [6]. This peptide, known as PSK, serves as a secreted disulfated pentapeptide [8]. On the other hand, PSK-β (the C-terminally truncated disulfated tetrapeptide “Tyr (SO3H)-Ile-Tyr (SO3H)-Thr”), while sharing similarities with PSK-α, may have distinct roles in specific plant species or developmental contexts, and its precise functions are still elusive [12,13]. In a notable experiment involving *Oryza sativa* OsPSK, the corresponding cDNA was genetically altered to encode the modified pentapeptide [12]. This specific modification was then isolated from the culture medium of transformed cells, providing concrete evidence that the transcript undergoes processing to generate the mature peptide. Interestingly, the culture medium also contained a C-terminally truncated tetrapeptide, indicating that PSK-β represents a degradation product originating from PSK-α.

The discovery of PSK’s biological activity originated from the purification of this peptide from conditioned medium extracted from a suspension culture of asparagus (*Asparagus officinalis* L.) [6]. Its isolation was based on its remarkable ability to induce cell division in asparagus mesophyll cells, especially when cultured at low cell density. The maturation process of PSK involves the enzymatic cleavage of its precursor proteins through proteolytic action, followed by post-translational sulfation, which is essential for attaining its full activity, as indicated by prior research [7,14]. Researchers have extensively explored the enzymes responsible for driving the biosynthesis of PSK. Notably, the subtilisin-like protease, SBT1.1, has emerged as a key player in this process, facilitating the proteolytic cleavage of PSK. It recognizes specific amino acid sequences, such as leucine and histidine, positioned upstream of the functional peptide domain [15]. The genes encoding these preproproteins are integral to the synthesis of active PSK peptides, which facilitate intercellular communication and influence numerous developmental pathways [14]. The multifunctional and versatile nature of PSK makes it a captivating subject of research, with implications not only for plant biology but also for potential applications in agriculture and biotechnology. It regulates cell growth [16], acts in the root apical meristem quiescent center cells [10,17], contributes to funicular pollen tube guidance [18], plays a crucial part in root development [10], is implicated in a plant’s response to environmental stressors, such as drought and salinity [19,20], and differentially changes immune responses depending on invaded pathogens [11,21]. It has been suggested that PSK integrates growth and defense signals to balance the competing metabolic costs of these responses [10,22,23].

Phytosulfokine receptors (PSKRs) are a class of integral transmembrane proteins characterized by their distinct extracellular domain and a C-terminal intracellular kinase domain. These receptors are classified as members of the receptor-like kinases, RLKs, which comprise a substantial number of representatives [12,16]. The PSKR proteins, which belong to a class of the leucine-rich repeat receptor-like kinase (LRR-RLK) family, exhibit a specific subcellular localization pattern. They are directed to the secretory pathway through the aid of an N-terminal signal peptide and securely anchored to the plasma membrane by means of a single transmembrane helix [24]. The function of PSKRs is of particular interest, and it has been established that they play a crucial role in mediating plant responses. Notably, *Os*PSKR15 has been shown to bind to a membrane fraction enriched in plasma membranes from rice suspension cells [25]. In Arabidopsis protoplasts, the expression of fusion proteins, like *At*PSKR1-GFP and *At*PSKR2-GFP, allowed for the visualization and confirmation of their presence at the plasma membrane [26].

PSK peptides interact with PSKRs to facilitate signal transduction, essential for plant development and defense. They significantly contribute to stimulating root development [14] and facilitating cell growth [27]. Additionally, PSKRs are crucial in mediating plant defense responses. Research in Arabidopsis has shown that PSK functions as a damage-associated molecular pattern, recognized primarily by *At*PSKR1, enhancing plant defenses against pathogen attacks [21]. In rice, *Os*PSKR1 has been associated with increased resistance to *Xanthomonas oryzae*, the pathogen responsible for bacterial leaf streak. This resistance is accompanied by the activation of pathogenesis-related genes linked to growth regulator pathways [28]. This dual role in development and defense underscores the importance of PSKRs in plant biology. PSKs are also essential for enhancing environmental stress tolerance. For instance, rice PSK signaling through its receptors regulates responses to various abiotic stresses, such as drought and salinity. The expression of *Os*PSKR15 under the drought stress condition indicates its role in stress signaling pathways [25]. Transgenic rice lines overexpressing specific PSK-related genes show improved drought tolerance, as evidenced by enhanced root development and increased biomass accumulation under water-limited conditions. Likewise, in Arabidopsis, the upregulation of PSK precursor genes in response to osmotic stress correlates with increased drought resilience. Research indicates that plants overexpressing PSK precursors exhibit superior growth and stress mitigation compared to wild-type plants [19]. These findings highlight the importance of PSK signaling in coordinating plant responses to several challenges.

In wheat, PSKRs are essential for growth, development, and various stress responses. Notably, the expression of the *TaPSKR1* gene was detected in roots, stems, leaves, and seeds and significantly elevated under drought and salt stress, indicating its essential role in helping wheat to cope with environmental stress [29]. In addition, the overexpression of *TaPSK5*-D, a modified version known as miR164-resistant TaPSK5-D, has been shown to enhance primary root growth and grain yield in rice [30]. The identification and characterization of *TaPSKRs* offer important insights into the role of PSK signaling in wheat, paving the way for further functional studies aimed at improving stress resilience and agronomic traits in this vital cereal crop.

The aim of this investigation was to comprehensively characterize the entire gene family of wheat phytosulfokine receptor genes and profile their expression across various wheat developmental stages. Through systematic analysis, all *PSKR* members were identified, assigned to wheat chromosomes, and annotated, providing insights into their structural features and functional attributes. In this study, the activity of *Ta*PSKR cDNAs was examined in RNA-seq datasets collected from various developmental phases. The potential interaction sites of PSKR receptors were also predicted through a docking screening analysis. This comprehensive characterization aimed to lay the foundation for future research on the role and regulation of *PSKRs* in wheat, offering valuable information for crop improvement and biotechnology applications.

## 2. Materials and Methods

### 2.1. TaPSKR Gene Family Identification

The latest released version of the *T. astevium* genome from IWGSC collected from the RefSeq v2.1 assembly (https://urgi.versailles.inrae.fr/download/iwgsc/IWGSC_RefSeq_Assemblies/v2.1/, accessed on 1 June 2023) and its annotation were used to search for *PSKRs*. The sequences of PSKR proteins collected from *T. aestivum* were used as queries to perform BLASTP searches against the wheat protein sequence database to find all putative PSKR proteins using BLASTP search. All candidates were manually verified with the conserved domain database to inspect the presence of their domains. By eliminating proteins that were not preserved in the latest genome version or, more likely, were considered to belong to another protein family, the PSKR gene family was collected. Genomic sequences, transcript sequences, and CDS sequences were all obtained. All *PSKR*s were analyzed by the EXPASy online tool (https://web.expasy.org/protparam/, accessed on 1 December 2023) to calculate the number of amino acids, molecular weight, and theoretical isoelectric points (pI). The subcellular localization of PSKR proteins was predicted using DeepLoc (https://services.healthtech.dtu.dk/service.php?DeepLoc-2.0, accessed on 1 December 2023 [31]), a tool specifically designed to determine protein localization based on their amino acid sequences. The MEME Suite version 5.5.0 (https://meme-suite.org/meme/, accessed on 1 December 2023 [32]) was utilized to identify and distribute ten conserved motifs within the CRK proteins. The functions of these identified motifs were analyzed using the ScanProsite viewer [33].

### 2.2. Chromosomal Localization and Nomenclature

Chromosomal localization of *PSKRs* in *T. aestivum* was mapped on respective chromosome sequences obtained from Wheat URGI (https://urgi.versailles.inra.fr/jbrowseiwgsc/gmod_jbrowse/?data=myData%2FIWGSC_RefSeq_v2.1&loc=Chr1A%3A239444899..359176993&tracks=DNA&highlight=, accessed on 1 January 2024) and represented using MapChart software (https://www.wur.nl/en/show/mapchart.htm, accessed on 1 January 2024). The *PSKRs* were designated in the order of their appearance on the chromosomes. In accordance with established guidelines for gene symbolization in *T. aestivum*, homologous genes in wheat were systematically named.

### 2.3. Phylogenetic Tree Construction

The *Ta*PSKR protein sequences were utilized to construct the phylogenetic tree to reveal their evolutionary relationships. The alignment of all protein sequences was performed using the CLUSTALW program within MEGA, version 11 (https://www.megasoftware.net/, accessed on 1 February 2024), focusing on sites that were 90% conserved [34]. Initial trees for the heuristic search were generated automatically by employing the neighbor-joining and BioNJ algorithms, based on a matrix of pairwise distances calculated using the JTT model [35]. The topology with the highest log-likelihood value was selected for further analysis. Subsequently, the phylogenetic tree was constructed using the maximum-likelihood method along with a JTT matrix-based model. The resulting tree is scaled, with branch lengths representing the number of substitutions per site. All positions containing gaps or missing data were excluded from the analysis.

### 2.4. RNA-Seq Data Collection

Publicly available RNA-seq data were sourced from the sequence read archive (SRA), a GenBank repository for high-throughput sequencing data that allows for extensive comparative analysis. Of the SRA databases, six RNA-seq experiments were to examine the transcript abundance of *TaPSKRs* across various developmental stages (Supplementary Material Table S1). The RNAseq datasets included RNA sequences collected from the first leaf of the 14-day-old seedling and the first flag leaves at 60 days of *T. aestivum* cultivar FLW30 and Toropi, respectively. All selected libraries were prepared in triplicate. These datasets were retrieved from two sources, including (1) BioProject PRJNA996310, released on 19 July 2023, by the ICAR-National Bureau of Plant Genetic Resources, and (2) BioProject PRJEB41456, released on 22 November 2021, by the European Bioinformatics Institute. Besides these, shoot and root RNA-seq datasets for wheat cultivars Chuanmai104 and Najran were also collected for analysis. These datasets, covering 14 and 21 days of wheat growth, were retrieved from BioProjects PRJNA925925 and PRJNA936261. The RNAseq dataset for Chuanmai104 was submitted by the Sichuan Academy of Agriculture Science on 20 January 2023, while the data for Najran was submitted by Newcastle University on 17 February 2023. The selected SRA datasets were paired-end Illumina sequences created by different platforms, including Illumina HiSeq 2000 and 4000 (San Diego, CA, USA), as well as NovaSeq 6000 (San Diego, CA, USA).

### 2.5. Quality Control

To guarantee the reliability and precision of the data, a rigorous quality control protocol for all collected data was implemented. The FastQC toolkit was utilized to assess the quality of the raw sequencing data, enabling the identification of low-quality sequences, overrepresented sequences, and adapter contamination [36]. Following this initial assessment, the Trimmomatic tool was employed to trim and filter out low-quality sequences and contaminants, ensuring the integrity of the data for downstream analyses [37].

### 2.6. Read Mapping

The high-quality short reads were aligned to the *Ta*PSKR reference sequences using Bowtie2 [38], which is known for its speed and accuracy in aligning sequencing reads to reference genomes. The filtered reads were subsequently mapped to the 61 *Ta*PSKR full-length cDNAs, and gene expression levels were quantified as transcripts per million reads (TPM) using StringTie [39], a method that normalizes for sequencing depth and gene length. Differential expression analysis of the TPM values was then performed using DESeq2, a widely adopted tool for this purpose [40], which employs a negative binomial generalized linear model to assess differential expression while accounting for data variability and controlling for multiple testing. This comprehensive analysis pipeline effectively facilitates the examination of gene expression patterns in the context of all *Ta*PSKRs.

### 2.7. Statistical Analysis and Visualization

Statistical analysis and heatmap visualizations in this study were performed using R version 4.3.0 (https://www.r-project.org/). *TaPSKRs* were considered differentially expressed if they met the following criteria: a false discovery rate (FDR) P-adjusted value < 0.05 and an absolute log2 fold change > 2. Heatmap figures were generated using the heatmap function with the pheatmap v1.0.12 package (https://cran.r-project.org/web/packages/pheatmap/index.html, accessed on 1 June 2024), enabling the visualization of complex patterns and relationships in gene expression [41].

### 2.8. Docking Screening Analysis

A single homeolog representative from the 25 *Ta*PSKR protein sequences, as well as the mature phytosulfokine type α (PSK-α) sequence comprising five amino acids (Tyr-Ile-Tyr-Thr-Gln), was utilized for secondary structure prediction. The Robetta server, a robust protein structure prediction tool, was employed to generate the most accurate models (https://robetta.bakerlab.org/ [42]). This modeling method resulted in a protein data bank (PDB) file for each protein to ensure high-quality structural predictions, facilitating further analysis of protein interactions.

Docking simulations were performed between each *Ta*PSKR protein and PSK-α using the LZerD web server (https://lzerd.kiharalab.org/ [43]). LZerD employs an efficient and reliable algorithm for protein–protein docking, allowing for the exploration of potential binding modes and interactions between the target proteins. The docking algorithm was run with default parameters, and a grid-based scoring function was utilized to evaluate the binding affinity of various protein conformations. This approach assesses structural compatibility and potential binding affinities between the proteins using evaluation statistics such as GOAP, DFIRE, and IT scores [44,45,46]. After generating docked complex models, the top 50,000 models, ranked by LZerD shape score, were selected and clustered using a root-mean-square deviation (RMSD) threshold of 5 Å. Cluster centers were then scored using the ranksum procedure described in the server scorer section. The top 10 models were advanced to the refinement protocol. The highest-ranking docking poses were selected for further analysis based on both energetic and geometric criteria.

## 3. Results

### 3.1. Genome-Wide Analysis of TaPSKR 

**Figure 1:**
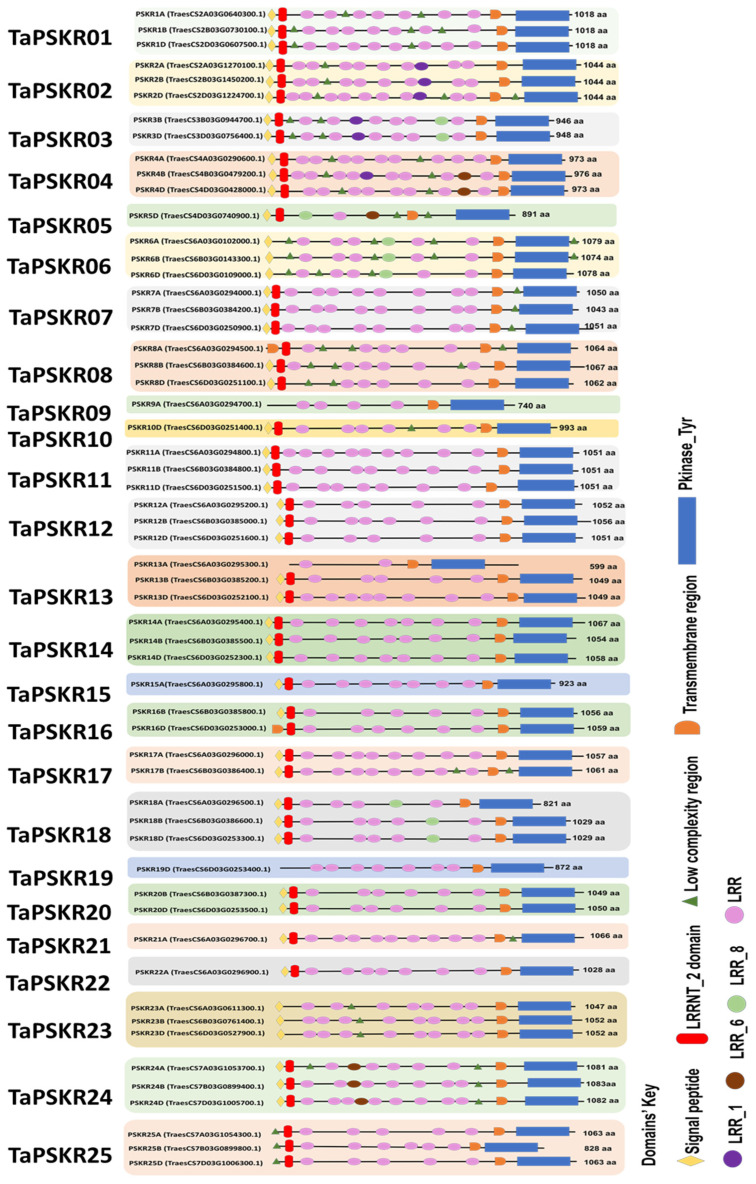
Wheat phytosulfokine receptors designated by their functional and structural domains. Twenty-five *Ta*PSKRs categorized by their domains are represented in a graph. The domains are labeled as follows: leucin-rich repeat (LRR, light mauve) with other subtypes LRR1 (dark mauve), LRR6 (brown), and LRR8 (green); leucine-rich repeat N-terminal 2 (LRRNT_2, red); the protein kinase-type tyrosine (PKinase_Tyr, blue); signal peptide (SP, yellow); transmembrane (TM, orange). The homeologs of *TaPSKRs* are depicted to highlight the variations within the family.

The comprehensive identification of the *PSKR* gene family in *T. aestivum*, utilizing the newly released reference genome v2.1 from the IWGSC, revealed 57 members distributed across the three subgenomes (A, B, and D), encompassing 25 distinct genes. All *TaPSKR* members and their designations are listed in Supplementary Material Table S2, and the coding sequences (CDSs) and protein sequences are collected in Data Files S1 and S2, respectively. The verification process involved a meticulous examination of these *Ta*PSKR proteins using conserved domain search tools such as SMART and InterproScan. This inspection confirmed the presence of key features characteristic of all *Ta*PSKR proteins, including the transmembrane domain (TM), at least one leucin-rich repeat (LRR), and protein kinase-type tyrosine (Pkinase_Tyr) (Figure 1). The majority of proteins possess both the leucine-rich repeat N-terminal 2 (LRRNT_2) domain and a signal peptide (SP). However, only a few proteins contain the LRR1, LRR6, and LRR8 domains. The diversification among these proteins was attributed to the number of LRR domains, which play vital roles in determining the structural and functional *Ta*PSKRs, impacting their binding capabilities and interaction with other molecular components [47]. All *Ta*PSKR proteins were scanned for conserved motifs using the MEME algorithm, resulting in the identification of 10 distinct motifs. The annotation of the identified motifs revealed several sites associated with post-translational modification features. Specifically, motifs 1, 5, 7, 8, 9, and 10 were found to contain the N-glycosylation site, while motifs 5 and 10 included the N-myristoylation site. Additionally, certain motifs were identified as phosphorylation sites: motif 6 contained cAMP- and cGMP-dependent protein kinase phosphorylation sites, and motifs 7 and 8 included casein kinase II phosphorylation sites (Supplementary Material Figure S1). The most significant function of the protein was identified in motif 3, which corresponds to the serine/threonine protein kinases’ active-site signature. This motif plays a crucial role in the functional activity of *Ta*PSKRs. The subcellular localization of *Ta*PSKR proteins was also performed using Deeploc. The analysis revealed their predicted localization on the cell membrane (Supplementary Material Table S2), suggesting their roles in signaling pathways.

**Figure 2:**
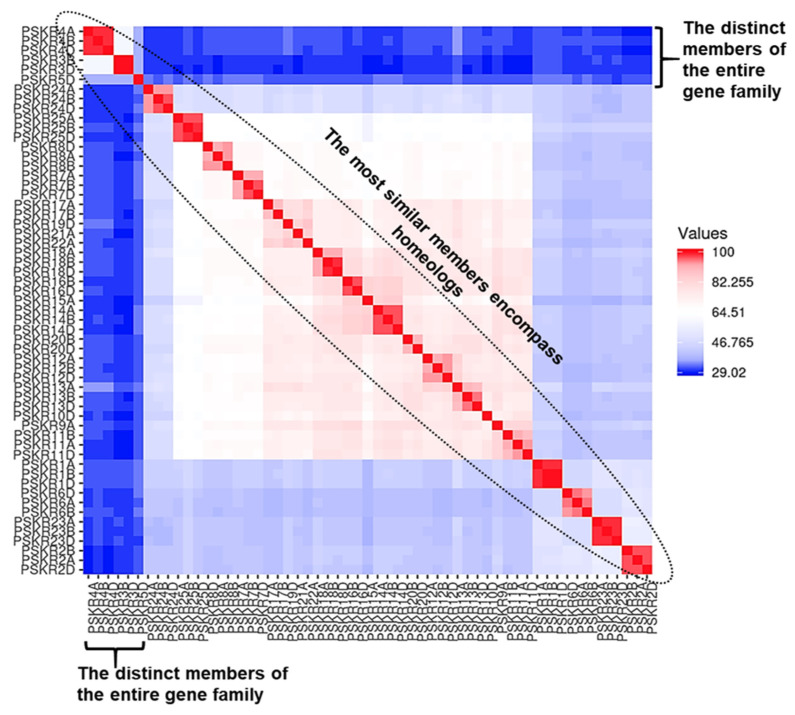
Heatmap depicting the pairwise protein sequence identity within all *Ta*PSKRs. The color gradient indicates differing levels of sequence identity, with red shades signifying higher protein sequence identity among proteins derived from homeologs. In contrast, blue shades represent the most divergent members to other members in the family, including *Ta*PSKR4A, *Ta*PSKR4B, *Ta*PSKR4D, *Ta*PSKR3B, *Ta*PSKR3D, and *Ta*PSKR5D.

*Ta*PSKR proteins exhibited substantial variability in their lengths, ranging from 599 to 1083 amino acids, and their molecular weights, spanning from 66.67 kDa in *Ta*PSKR13A to 115.41 kDa in *Ta*PSKR24B. Additionally, the isoelectric points (pI) of these proteins fell within the range of 5.5 to 8.55, providing valuable insights into their biochemical characteristics. The highest protein sequence identity among all *Ta*PSKRs was observed between the homeologs *Ta*PSKR4B and *Ta*PSKR4D (98.36%), while the lowest identity was recorded at 29.02% between the paralogs *Ta*PSKR3D and *Ta*PSKR11B (Supplementary Material Table S2). The heatmap illustrates the protein sequence identity within the all designated *Ta*PSKRs, using a color gradient to represent varying levels of sequence similarity (Figure 2). Red shades indicate higher protein identity among homeologs, while blue shades denote the most divergent members to other members of the family, which include TaPSKR3B, *Ta*PSKR3D, *Ta*PSKR4A, *Ta*PSKR4B, *Ta*PSKR4D, and *Ta*PSKR5D.

### 3.2. Chromosomal Assignment of TaPSKR 

**Figure 3:**
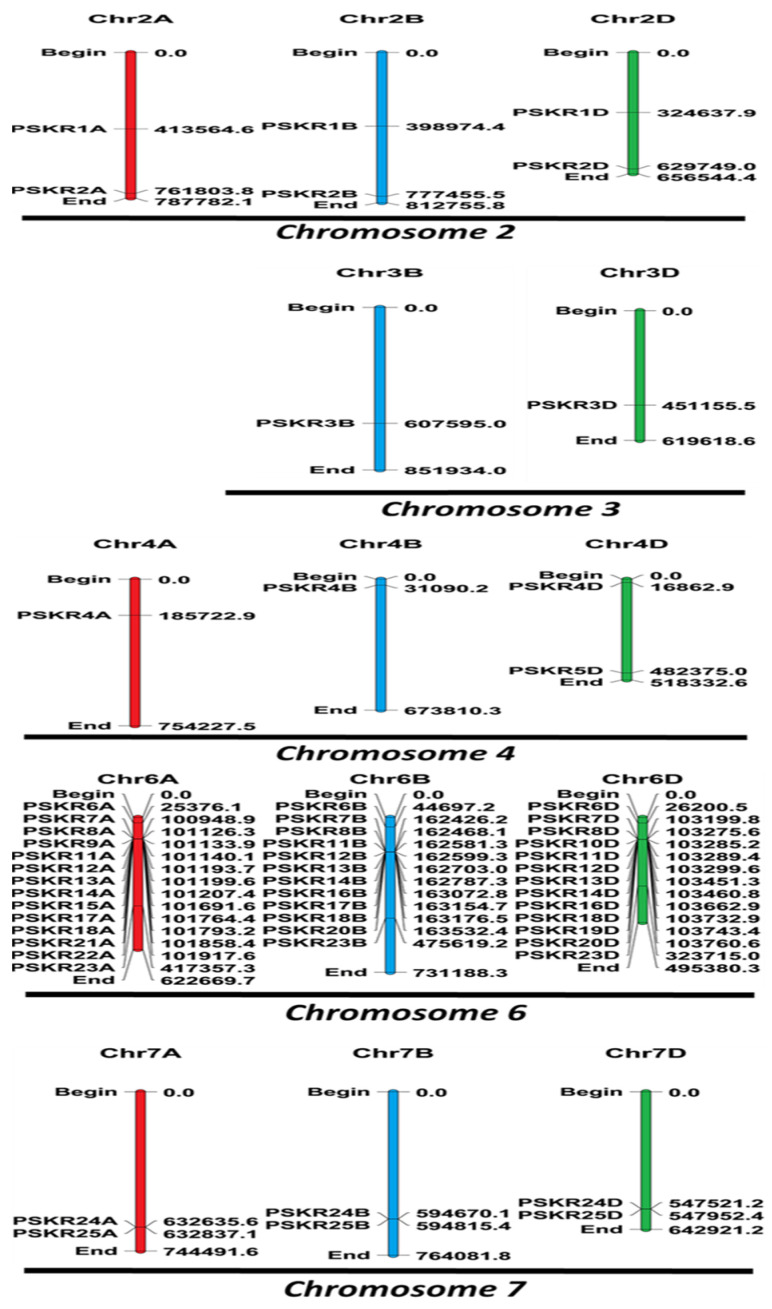
Distribution of *TaPSKRs* across all wheat chromosomes. Heterogeneous distribution of 25 *TaPSKRs*, exhibiting significant variation. The subgenomes of chromosomes 1 and 5, as well as chromosome 3A, were found to be devoid of any *TaPSKR* gene members, while chromosome 6 displayed the highest abundance, encompassing a total of 39 members. The chromosomes from subgenomes A, B, and D are highlighted in red, blue, and green, respectively.

The assignment of chromosomal localization to the *PSKR* gene family adds a layer of insight into the genomic distribution of these genes in *T. aestivum*. This study revealed a non-uniform distribution of the 25 *TaPSKRs* across the chromosomes, highlighting significant variations in gene chromosomal assignment. Notably, the three subgenomes of chromosomes 1 and 5 were devoid of any detected *TaPSKR* gene members, as was the subgenome of chromosome 3A (Figure 3). Conversely, chromosome 6 was found to harbor the highest number of *TaPSKR* gene members, with a total of 39 members distributed among its three subgenomes. When designating the *PSKR*s to their respective homeologs in *T. aestivum*, the established gene nomenclature guidelines for this species were strictly followed [48]. By consistently applying these naming principles, the *TaPSKRs* in wheat can be easily recognized, and their evolutionary relationships can be inferred.

### 3.3. Evolution of TaPSKR Gene Family

**Figure 4:**
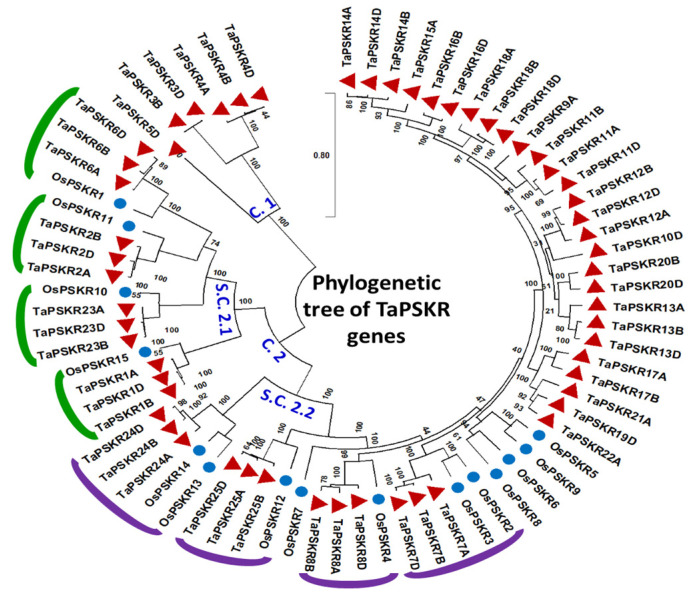
Phylogenetic tree of wheat *PSKR* members and their rice orthologs. Initial trees for the heuristic search were generated automatically by employing the neighbor-joining and BioNJ algorithms. Subsequently, the phylogenetic tree was constructed using the maximum-likelihood method along with a JTT matrix-based model. The numbers at nodes represents bootstrap values per 1000 replicates. Two main clusters were identified. Cluster 1 (C.1) includes the most distantly related *TaPSKR* members, comprising the three homeologs of gene 4 (*TaPSKR4A*, *TaPSKR4B*, *TaPSKR4D*), as well as *TaPSKR3D* and *TaPSKR5D*. Cluster 2 (C.2) is further divided into two subclusters: Subcluster C.2.1 consists of genes paired with rice orthologs, including *TaPSKR6*, *TaPSKR2*, *TaPSKR23*, and *TaPSKR1* matched with *OsPSKR1*, OsPSKR11, *OsPSKR10*, and *OsPSKR15*, respectively. Subcluster C.2.2 contains a mix of rice genes and predominantly wheat phytosulfokine genes without rice orthologs. Red triangles represent *TaPSKR* members. Blue circles represent *OsPSKR* members.

The exploration of the evolutionary relationship within the wheat *PSKR* gene family has provided valuable insights into their diversification and functional roles across the evolutionary history of wheat. By constructing a phylogenetic tree in conjunction with rice phytosulfokine receptors (*OsPSKRs*) generated by Nagar et al. [49], the relationships among *TaPSKR* members were assessed. The multiple sequence alignment of both wheat and rice *PSKR*s (*TaPSKRs* and *OsPSKRs*) unveiled a distribution pattern, emphasizing significant diversification within the gene family. The generated phylogenetic tree revealed the presence of two key clusters (Figure 4). The first cluster (C.1) encompassed the most distant *TaPSKR* members to the other genes. This cluster included the three homeologs of gene 4 (*TaPSKR4A*, *TaPSKR4B*, and *TaPSKR4D*). Additionally, it contained *TaPSKR3B* and *TaPSKR3D*, as well as the single representative of gene 5, *TaPSKR5D*. The second cluster (C.2) comprised two subclusters. The C.2.1 subcluster included four genes, the three homeologs of *TaPSKR6*, *TaPSKR2*, *TaPSKR23*, and *TaPSKR1*, paired with rice orthologs, *OsPSKR1*, *OsPSKR11*, *OsPSKR10*, and *OsPSKR15*, respectively. Furthermore, the S.C.2.2 subcluster included the remaining rice genes, but the majority of the subcluster was composed of wheat phytosulfokine receptor genes that did not have rice orthologs.

### 3.4. Expression Profile of the TaPSKRs 

**Figure 5:**
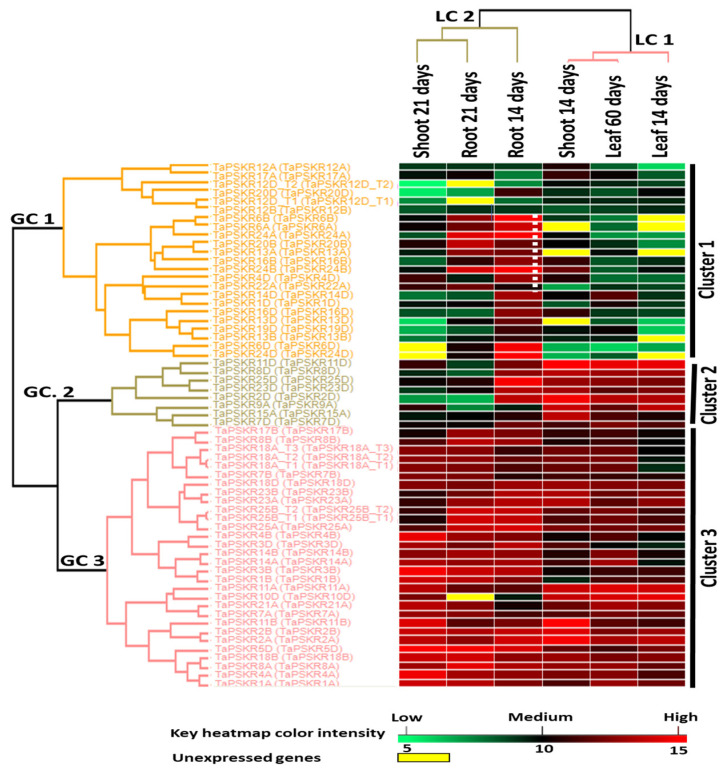
Heatmap of *TaPSKR* gene expression across various developmental stages of wheat. The heatmap illustrates the abundance of sixty-one *Ta*PSKR transcripts quantified from six RNA-seq libraries representing wheat at developmental stages of 14, 21, and 60 days, including first seedling and flag leaf, shoot, and root tissues. The expression levels of *TaPSKRs* were normalized using transcripts per million (TPM), and the log2 values of these TPM measurements were calculated. In the heatmap, red indicates high gene expression levels, while green represents low expression levels. Genes with moderate expression are shown in black, and unexpressed genes are marked in yellow. The white dotted line highlights genes that are exclusively highly expressed in root libraries.

The expression of the *TaPSKR* gene family was profiled. The 61 *Ta*PSKR cDNAs were found to be abundantly represented across multiple RNA-seq libraries, which were derived from diverse wheat cultivars and developmental stages. To analyze the expression of the *TaPSKR* gene family, six different RNA-seq libraries previously generated and publicly available in the GenBank sequence read archive (SRA) represented in triplicates were utilized. These libraries were derived from various temporal and spatial stages of wheat development, including plants at 14, 21, and 60 days old, as well as diverse tissues such as the first seedling and flag leaves, shoots, and roots. By leveraging this comprehensive set of RNA-seq data, the expression profile of the *Ta*PSKR transcripts that are represented in transcript per million (TPM) is listed in Supplementary Material Table S3 and graphed in histograms (Supplementary Material Figure S2). This approach enabled a broad survey of *TaPSKR* gene abundance in wheat, laying the groundwork for further targeted experiments to elucidate the specific functions of this gene family in wheat growth and development. Based on the relative expression levels of *TaPSKRs* across normalized libraries, the hierarchical clustering of the *TaPSKR* gene family resulted in the formation of three major clusters (Figure 5). The first gene cluster (GC 1) specifically highlighted genes partially associated with root development. Within this cluster, several genes exhibited high to moderate expression levels in root tissues, including *TaPSKR6B*, *TaPSKR6A*, *TaPSKR24A*, *TaPSKR20B*, *TaPSKR13A*, *TaPSKR16B*, *TaPSKR24B*, *TaPSKR4D*, and *TaPSKR22A*. The second cluster (GC 2) encompassed genes that exhibited high expression at all stages of tested leaves, as well as in shoots and roots at 14 days. However, their expression was moderate to low in libraries derived from 21-day-old shoots and roots. The third cluster (GC 3) comprised *TaPSKR*s that were constitutively expressed across all tested libraries, with the exception of *TaPSKR10D*, which remained unexpressed in this group. The consistently high expression levels of these genes across various tissues and developmental stages highlight their importance in fundamental cellular processes and development.

The hierarchical clustering analysis of the *TaPSKR* gene expression data revealed two key clusters based on the Ta*PSKR* expression profiles across the tested libraries (Figure 5). The first library cluster (LC 1) comprised libraries from leaves and shoots at the ages of 14 and 60 days, indicating a strong association between these tissues and developmental stages. In contrast, the second library cluster (LC 2) included all root libraries and the shoots at 21 days, suggesting distinct expression patterns in these tissues as they mature. Additionally, pairwise correlation analysis conducted among the tested libraries demonstrated similar expression behaviors, as illustrated in Supplementary Material Figure S3. Notably, a remarkably high Pearson correlation of 0.824 was observed between the first seedling and flag leaves at 14 and 60 days, respectively. This was followed by a correlation of 0.756 between the leaf and shoot libraries at 14 days and a correlation of 0.751 between the shoot and leaf libraries at 14 and 60 days.

### 3.5. Molecular Docking Simulations of Phytosulfokines and Phytosulfokine Receptors

**Figure 6:**
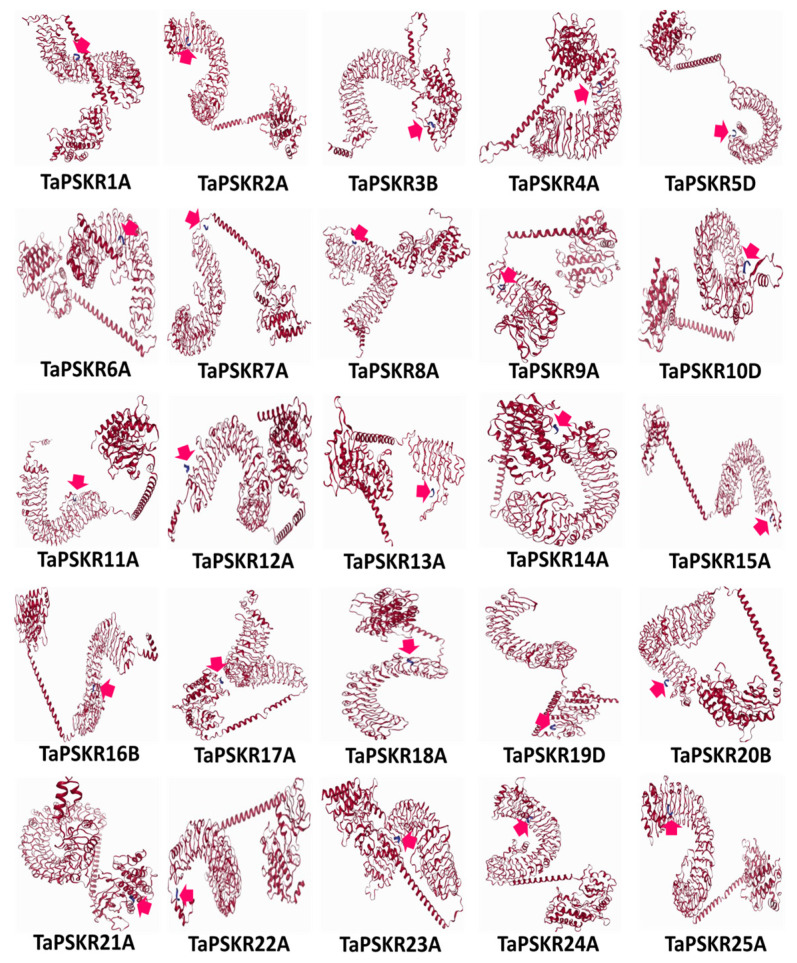
Molecular docking of phytosulfokine–phytosulfokine receptor interactions. The 2D interaction graph illustrates the protein complex formed between each phytosulfokine receptor and the phytosulfokine type α protein’s secondary structure. In the graph, red indicates phytosulfokine receptors, while blue represents phytosulfokine. The pink arrow indicates the position of the interaction.

In this investigation, the molecular docking web server LZerD was employed to evaluate the binding affinity and interactions between the mature sequence of PSK-α and a representative homeolog for each gene of 25 *Ta*PSKR protein sequences. Notably, the majority of the *Ta*PSKR proteins exhibited interactions with PSK within the LRR region (Figure 6). For this, the secondary structure of the mature PSK-α protein, which comprises five specific amino acids (Tyr-Ile-Tyr-Thr-Gln) out of a total of eighty amino acids in the full-length protein, was generated. Furthermore, the secondary structures of all 25 wheat PSKR proteins were modeled using Robetta to ensure high accuracy. The optimal protein models were selected based on their maximum sequence identities and coverage. These receptor protein models were subsequently assessed against PSK using the LZerD algorithm in pairwise docking mode. Most *Ta*PSKR proteins were found to interact with PSK specifically in the LRR region (Figure 6). Rankings of the docking simulation results were established using GOAP, DFIRE, and IT scores, which were compiled into a final ranking (Supplementary Material Table S4) to provide a comprehensive evaluation of the docking affinities among the tested proteins. This analysis indicated that PSK-α consistently yielded the most favorable scores when interacting with *Ta*PSKR1A, *Ta*PSKR3B, and *Ta*PSKR13A, thereby corroborating the observed docking model rankings.

## 4. Discussion

In the era of agricultural innovation, leveraging extraordinary biomolecules proves promising for enhancing wheat development and resilience to diverse challenges. One captivating instance of an extraordinary biomolecule in this context is phytosulfokine (PSK), a pentapeptide hormone with pivotal roles in plant growth and response to severe conditions. PSK receptors (PSKRs) emerge as integral players in plant cellular communication, characterized by their distinctive extracellular and intracellular domains within the RLK family [12,16]. The established role of PSKRs in mediating plant development and responses is universally recognized, evident in their binding to plasma membrane fractions across diverse plant species [8,10,24]. The unique structural features and conserved functionality of PSKRs underscore their pivotal role in deciphering and transducing extracellular signals in plant cells, offering a promising avenue for further exploration into the intricate realm of plant molecular biology. Despite the importance of PSK signaling pathways, there has been limited exploration of the specific functions and regulatory mechanisms of PSKRs within this key crop species. The investigation aimed to fill existing knowledge gaps and elucidate the functional roles of these genes. The findings provide a robust foundation for exploring the gene structure, functional attributes, and regulatory roles of the *PSKR*. This research opens up promising avenues for crop improvement and biotechnological applications in wheat.

In this investigation, I provided insights into the *PSKR* gene family in *Triticum aestivum* L., revealing their structural, functional, and evolutionary characteristics. The comprehensive identification of 57 *TaPSKR* members across the three wheat subgenomes (A, B, and D) underscores the complexity and diversity of this gene family, with 25 distinct genes verified through conserved domain analysis. The presence of critical features such as TM, LRRs, and Pkinase domains confirms the functional relevance of these proteins in signaling pathways [13]. The variation among *Ta*PSKR proteins is primarily attributed to the differing number of LRR domains they contain. These LRR domains play a crucial role in determining the structural and functional diversity of the proteins, influencing their binding capabilities, stability, and interaction with other molecular components [47]. Moreover, the identification of post-translational modification sites, including N-glycosylation and phosphorylation, further emphasizes the regulatory complexity of these proteins and their potential roles in modulating signaling pathways [50]. Studying the protein sequence identity of *Ta*PSKRs provides valuable information about the evolutionary connections within the *TaPSKR* gene family. The homeologs TaPSKR4B and TaPSKR4D have a protein sequence identity of 98.36%, suggesting significant conservation and possible functional overlap. On the other hand, the significant difference of 29.02% in identity between the paralogs *Ta*PSKR3D and *Ta*PSKR11B indicates a considerable level of divergence. All these designated features highlight the functional complexity of these receptors, which are predicted to play critical roles in plant signaling pathways and development.

This study also adhered to established gene nomenclature guidelines [48], facilitating the recognition and comparative analysis of *PSKRs* across *T. aestivum*. The chromosomal distribution of the *TaPSKRs* reveals a non-uniform allocation, with certain chromosomes lacking any Ta*PSKR* members. Notably, all subgenomes of chromosomes 1 and 5 as well as 3A were devoid of any *TaPSKR* gene members, while chromosome 6 contained the highest concentration, housing 39 members. This non-uniform distribution suggests potential evolutionary pressures influencing gene retention and loss [51]. This pattern prompts further investigation into the genetic and evolutionary mechanisms that dictate the presence or absence of these genes on specific chromosomes.

Phylogenetic analysis indicates substantial diversification within the *TaPSKR* gene family, with the formation of distinct clusters that reflect evolutionary relationships. The high sequence identity observed among some homeologs, contrasted with significant divergence in others, suggests a dynamic evolutionary history that has shaped the functional roles of these receptors. By constructing a phylogenetic tree alongside rice phytosulfokine receptors (*OsPSKRs*) as established by Nagar et al. [49], two distinct clusters were identified within the TaPSKR gene family, highlighting the substantial diversification that has occurred in three key clusters. The first cluster (C.1) included the most distantly related *TaPSKR* members, particularly the homeologs of *TaPSKR3, TaPSKR4*, and *TaPSKR5*, indicating a divergence that may reflect adaptation to specific functions [52]. In contrast, the C.2 cluster was further divided into two subclusters, with the C.2.1 subcluster containing genes closely associated with rice orthologs, suggesting conserved functional roles across species. The presence of wheat-specific *TaPSKRs* in the C.2.2 subcluster, which lacked rice counterparts, implies unique evolutionary adaptations within the wheat lineage. The exploration of the evolutionary relationships within the TaPSKR gene family has unveiled significant insights into their diversification and functional roles throughout the evolutionary history of wheat. Overall, these findings provide a foundational understanding of the evolutionary trajectory of the *TaPSKR* gene family, paving the way for future functional studies aimed at elucidating their roles in plant growth and development.

Expression profiling of Ta*PSKR* across various developmental stages and tissues highlights valuable insights into their functional roles during wheat development. By leveraging a comprehensive set of RNA-seq data derived from diverse wheat cultivars and developmental stages, the expression patterns of 61 TaPSKR transcripts were examined across multiple growth stages. Hierarchical clustering of the TaPSKR gene expression data revealed three major clusters (GC 1, GC 2, and GC 3). The GC 1 cluster highlighted genes partially associated with root development, with several members exhibiting high to moderate expression levels in root tissues. This includes *TaPSKR6B, TaPSKR6A, TaPSKR24A, TaPSKR20B, TaPSKR13A, TaPSKR16B, TaPSKR24B, TaPSKR4D,* and *TaPSKR22A*. The significant abundance of these genes in root tissues underscores their functional specificity and suggests that they play critical roles in regulating root growth and development compared to their expression in other tissues. In a former investigation, the overexpression of *TaPSK5D* revealed enhancement of primary root growth and grain yield in rice [30]. The identification and characterization of *TaPSKRs* exclusively expressed in roots may offer important insights into the role of PSK signaling in root development, which is vital to helping plants adapt to drought challenges. The GC 2 cluster encompassed genes exhibiting high expression levels in leaves at 14 and 60 days, as well as in shoots and roots at 14 days. This expression pattern implies that these *TaPSKRs* may play crucial roles during specific developmental seedling stages. The last cluster, GC 3, comprised *TaPSKRs* that were constitutively expressed across all tested libraries, with the exception of *TaPSKR10D*. The ubiquitous presence of these TaPSKR transcripts suggests that they serve as essential regulators of growth and development throughout the wheat life cycle [49]. Pairwise correlation analysis among the tested libraries demonstrated similar expression behaviors. Notably, a remarkably high Pearson correlation of 0.824 was observed between the first seedling and flag leaves at 14 and 60 days, respectively. This finding indicates a significant level of expression similarity among specific tissue types and developmental stages, highlighting the potential regulatory roles of the *TaPSKRs* in wheat development.

The molecular docking simulations demonstrate the binding affinity of PSK-α with *Ta*PSKR proteins, reinforcing the importance of these interactions in the signaling mechanisms of wheat. The favorable docking scores for certain receptor models indicate their potential as key players in the PSK signaling pathway. By utilizing the molecular docking web server LZerD, assessment of the binding affinities and interactions between the mature sequence of PSK-α and a representative homeolog from each of the 25 homologous PSKR protein sequences highlighted the potential for specific receptor–ligand interactions. The docking results revealed that the majority of the *Ta*PSKR proteins interacted with PSK-α within the leucine-rich region, a domain known for its role in protein–protein interactions and signaling. This finding is consistent with the established functions of LRR domains in mediating receptor–ligand interactions, suggesting that these regions are critical for the binding and subsequent signaling activities of PSKRs. The vital role of LRR in signal recognition was reviewed by [53]. The variability in the number of LRR domains among TaPSKRs is particularly noteworthy, as these domains are integral to the structural and functional diversity of the receptors. This diversity likely influences their binding capabilities and interactions with the PSK, which are crucial for plant growth and development. The specific interactions observed between PSK-α and key receptor proteins, such as *Ta*PSKR1A, *Ta*PSKR3B, and *Ta*PSKR13A, indicate that these receptors may play pivotal roles in the PSK signaling pathway, potentially influencing various developmental processes in wheat. Furthermore, the implications of these findings extend beyond mere binding affinities. The identification of specific receptor proteins that exhibit strong interactions with PSK-α opens avenues for further research into the downstream signaling pathways activated by these interactions. The investigations into PSKRs add complexity to the study of plant signaling [6,7,12,16,54]. Understanding the intricate role of PSKRs lays the groundwork for future research in wheat genetics. In addition, deciphering how PSK-α binds to its receptors and the subsequent cellular responses can provide insights into the regulatory mechanisms governing plant growth and development.

## 5. Conclusions

The comprehensive analysis of the *T. aestivum* PSKR reveals significant structural, functional, and evolutionary insights. With 57 identified members across three subgenomes and 25 distinct genes confirmed, this study highlights the complexity and diversity of the gene family. Variations in LRR domains underscore the functional relevance of these receptors in plant signaling. The non-uniform chromosomal distribution suggests evolutionary pressures at play, while phylogenetic analysis indicates substantial diversification and distinct clusters within the *TaPSKR* gene family. Expression profiling of *TaPSKRs* across wheat developmental stages and tissues provides insights into their functional roles. RNA-seq data analysis identified three major expression clusters. The GC 1 cluster was partially associated with root development, suggesting that specific *TaPSKRs* regulate root growth, particularly in response to drought. The GC 2 cluster highlighted genes with high expression in leaves at all selected stages, as well as shoots and roots during early seedling development. Finally, GC 3 consisted of constitutively expressed genes, emphasizing their essential regulatory functions throughout the wheat life cycle. Molecular docking simulations demonstrated PSK-α’s binding affinity with specific *Ta*PSKR proteins, reinforcing their role in PSK signaling pathways. Interactions between PSK-α and *Ta*PSKRs, particularly in LRR domains, are crucial for mediating developmental processes. Overall, these findings lay the groundwork for future research into the roles of PSKRs in plant growth and development, offering potential avenues for enhancing wheat resilience and productivity through biotechnological applications.

## 6. Perspectives

The comprehensive identification of the phytosulfokine receptor in wheat has laid a strong foundation for future research aimed at leveraging biotechnological approaches to modify plant phenotypes and enhance wheat resilience to environmental challenges. The insights gained from this study, particularly the association of specific *TaPSKRs* with root development, open up exciting avenues for targeted crop improvement strategies. One promising avenue for future research is the generation of transgenic wheat lines overexpressing key *TaPSKRs* identified in this study as being crucial for root development. By stimulating the production of PSK and manipulating the receptors’ perception, it may be possible to engineer wheat cultivars with enhanced root systems that are better equipped to cope with drought stress. The overexpression of *TaPSK5D*, which has been shown to enhance primary root growth and grain yield in rice [30], serves as a proof of concept for this approach. Furthermore, the role of phytosulfokine peptides in disease resistance remains an intriguing area for future investigation. While the current study has primarily focused on the developmental aspects of PSKR signaling, it is conceivable that these receptors may also play a role in mediating wheat plant defense responses against pathogens. This may lead to the identification of key receptors that are essential for enhancing pathogen-related genes (PRs) that may be activated during the PSK signal transduction processes.

## Data Availability

The original contributions presented in the study are included in the article and Supplementary Materials, further inquiries can be directed to the author.

## Supplementary Materials

The following supporting information can be downloaded at https://www.mdpi.com/article/10.3390/genes15101306/s1: Data File S1: The coding region (CDS) sequences of the *T. aestivum* PSKR gene family; Data File S2: The protein sequences of the *T. aestivum* PSKR gene family; Figure S1: Identification and characterization of conserved motifs in the *Ta*PSKR proteins; Figure S2: Histogram depicting the transcript expression levels of Ta*PSKR*s mapped to selected RNA-seq libraries; Figure S3: Pearson pairwise correlation of the expression patterns of *TaPSKRs* at various stages; Table S1: Libraries used for studying the expression of the *TaPSKR*s; Table S2: Identification, characterization, and localization of 57 members of the phytosulfokine proteins in *T. aestivum*; Table S3: The relative transcript expression level of Ta*PSKR*s normalized by transcripts per million (TPM); Table S4: Predicted interaction sites and affinity scores between phytosulfokine and phytosulfokine receptors using lzerd.

## References

[1] Viana C.M., Freire D., Abrantes P., Rocha J., Pereira P. (2022). Agricultural land systems importance for supporting food security and sustainable development goals: A systematic review. Sci. Total Environ..

[2] Roychowdhury R., Ballén-Taborda C., Chaturvedi P. (2023). Characterizing and improving traits for resilient crop development. Front. Plant Sci..

[3] Borisjuk N., Kishchenko O., Eliby S., Schramm C., Anderson P., Jatayev S., Kurishbayev A., Shavrukov Y. (2019). Genetic Modification for Wheat Improvement: From Transgenesis to Genome Editing. BioMed Res. Int..

[4] Ijaz M., Khan F., Ahmed T., Noman M., Zulfiqar F., Rizwan M., Chen J., Siddique K., Li B. (2023). Nanobiotechnology to advance stress resilience in plants: Current opportunities and challenges. Mater. Today Bio.

[5] Waadt R., Seller C.A., Hsu P.K., Takahashi Y., Munemasa S., Schroeder J.I. (2022). Plant hormone regulation of abiotic stress responses. Nat. Rev. Mol. Cell Biol..

[6] Matsubayashi Y., Sakagami Y. (1996). Phytosulfokine, sulfated peptides that induce the proliferation of single mesophyll cells of *Asparagus officinalis* L.. Proc. Natl. Acad. Sci. USA.

[7] Komori R., Amano Y., Ogawa-Ohnishi M., Matsubayashi Y. (2009). Identification of tyrosylprotein sulfotransferase in Arabidopsis. Proc. Natl. Acad. Sci. USA.

[8] Matsubayashi Y. (2014). Posttranslationally modified small-peptide signals in plants. Annu. Rev. Plant Biol..

[9] Sauter M. (2015). Phytosulfokine peptide signalling. J. Exp. Bot..

[10] Stührwohldt N., Dahlke R.I., Steffens B., Johnson A., Sauter M. (2011). Phytosulfokine-α controls hypocotyl length and cell expansion in *Arabidopsis thaliana* through phytosulfokine receptor 1. PLoS ONE.

[11] Mosher S., Seybold H., Rodriguez P., Stahl M., Davies K.A., Dayaratne S., Morillo S.A., Wierzba M., Favery B., Keller H. (2013). The tyrosine-sulfated peptide receptors PSKR1 and PSY1R modify the immunity of Arabidopsis to biotrophic and necrotrophic pathogens in an antagonistic manner. Plant J..

[12] Yang H., Matsubayashi Y., Nakamura K., Sakagami Y. (1999). *Oryza sativa* PSK gene encodes a precursor of phytosulfokine-Alpha, a sulfated peptide growth factor found in plants. Proc. Natl. Acad. Sci. USA.

[13] Li Y., Di Q., Luo L., Yu L. (2024). Phytosulfokine peptides, their receptors, and functions. Front. Plant Sci..

[14] Kutschmar A., Rzewuski G., Stührwohldt N., Beemster G.T.S., Inzé D., Sauter M. (2009). PSK-α promotes root growth in Arabidopsis. New Phytol..

[15] Srivastava R., Liu J.X., Howell S.H. (2008). Proteolytic processing of a precursor protein for a growth-promoting peptide by a subtilisin serine protease in Arabidopsis. Plant J..

[16] Ladwig F., Dahlke R.I., Stührwohldt N., Hartmann J., Harter K., Sauter M. (2015). Phytosulfokine Regulates Growth in Arabidopsis through a Response Module at the Plasma Membrane That Includes CYCLIC NUCLEOTIDE-GATED CHANNEL17, H+-ATPase, and BAK1. Plant Cell.

[17] Heyman J., Cools T., Vandenbussche F., Heyndrickx K.S., Van Leene J., Vercauteren I., Vanderauwera S., Vandepoele K., De Jaeger G., Van Der Straeten D. (2013). ERF115 controls root quiescent center cell division and stem cell replenishment. Science.

[18] Stührwohldt N., Dahlke R.I., Kutschmar A., Peng X., Sun M.X., Sauter M. (2015). Phytosulfokine peptide signaling controls pollen tube growth and funicular pollen tube guidance in Arabidopsis thaliana. Physiol. Plant..

[19] Stührwohldt N., Bühler E., Sauter M., Schaller A. (2021). Phytosulfokine (PSK) precursor processing by subtilase SBT3.8 and PSK signaling improve drought stress tolerance in Arabidopsis. J. Exp. Bot..

[20] Saini S., Kaur N., Marothia D., Singh B., Singh V., Gantet P., Pati P.K. (2021). Morphological Analysis, Protein Profiling and Expression Analysis of Auxin Homeostasis Genes of Roots of Two Contrasting Cultivars of Rice Provide Inputs on Mechanisms Involved in Rice Adaptation towards Salinity Stress. Plants.

[21] Igarashi D., Tsuda K., Katagiri F. (2012). The peptide growth factor, phytosulfokine, attenuates pattern-triggered immunity. Plant J..

[22] Amano Y., Tsubouchi H., Shinohara H., Ogawa M., Matsubayashi Y. (2007). Tyrosine-sulfated glycopeptide involved in cellular proliferation and expansion in Arabidopsis. Proc. Natl. Acad. Sci. USA.

[23] Hartmann J., Stührwohldt N., Dahlke R.I., Sauter M. (2013). Phytosulfokine control of growth occurs in the epidermis, is likely to be non-cell autonomous and is dependent on brassinosteroids. Plant J..

[24] Matsubayashi Y., Ogawa M., Morita A., Sakagami Y. (2002). An LRR receptor kinase involved in perception of a peptide plant hormone, phytosulfokine. Science.

[25] Nagar P., Sharma N., Jain M., Sharma G., Prasad M., Mustafiz A. (2022). OsPSKR15, a phytosulfokine receptor from rice enhances abscisic acid response and drought stress tolerance. Physiol. Plant..

[26] Hartmann J., Linke D., Bönniger C., Tholey A., Sauter M. (2015). Conserved phosphorylation sites in the activation loop of the Arabidopsis phytosulfokine receptor PSKR1 differentially affect kinase and receptor activity. Biochem. J..

[27] Matsubayashi Y., Ogawa M., Kihara H., Niwa M., Sakagami Y. (2006). Disruption and Overexpression of Arabidopsis Phytosulfokine Receptor Gene Affects Cellular Longevity and Potential for Growth. Plant Physiol..

[28] Yang W., Zhang B., Qi G., Shang L., Liu H., Ding X., Chu Z. (2019). Identification of the phytosulfokine receptor 1 (OsPSKR1) confers resistance to bacterial leaf streak in rice. Planta.

[29] Zhang P.P., Chen T., Jing F.L., Liu Y., Ma J.F., Tian T., Wang P., Yang D.L. (2023). Cloning and expression analysis of TaPSKR1 gene in wheat. Acta Agric. Bor. Sin..

[30] Geng Y., Jian C., Xu W., Liu H., Hao C., Hou J., Liu H., Zhang X., Li T. (2020). miR164-targeted TaPSK5 encodes a phytosulfokine precursor that regulates root growth and yield traits in common wheat (*Triticum aestivum* L.). Plant Mol. Biol..

[31] Thumuluri V., Almagro Armenteros J.J., Johansen A.R., Nielsen H., Winther O. (2022). DeepLoc 2.0: Multi-label subcellular localization prediction using protein language models. Nucleic Acids Res..

[32] Bailey T.L., Johnson J., Grant C.E., Noble W.S. (2015). The MEME Suite. Nucleic Acids Res..

[33] de Castro E., Sigrist C.J., Gattiker A., Bulliard V., Langendijk-Genevaux P.S., Gasteiger E., Bairoch A., Hulo N. (2006). ScanProsite: Detection of PROSITE signature matches and Pro Rule-associated functional and structural residues in proteins. Nucleic Acids Res..

[34] Tamura K., Stecher G., Kumar S. (2021). MEGA11: Molecular Evolutionary Genetics Analysis Version 11. Mol. Biol. Evol..

[35] Jones D.T., Taylor W.R., Thornton J.M. (1992). The rapid generation of mutation data matrices from protein sequences. Comput. Appl. Biosci..

[36] Andrews S. (2010). FastQC: A Quality Control Tool for High Throughput Sequence Data.

[37] Bolger A.M., Lohse M., Usadel B. (2014). Trimmomatic: A flexible trimmer for Illumina sequence data. Bioinformatics.

[38] Langmead B., Salzberg S.L. (2012). Fast gapped-read alignment with Bowtie 2. Nat. Methods.

[39] Pertea M., Pertea G.M., Antonescu C.M., Chang T.C., Mendell J.T., Salzberg S.L. (2015). StringTie enables improved recon-struction of a transcriptome from RNA-seq reads. Nat. Biotechnol..

[40] Love M.I., Huber W., Anders S. (2014). Moderated estimation of fold change and dispersion for RNA-seq data with DESeq2. Genome Biol..

[41] Dessau R.B., Pipper C.B. (2008). “R”—Project for statistical computing. Ugeskr. Laeger.

[42] Baek M., DiMaio F., Anishchenko I., Dauparas J., Ovchinnikov S., Lee G.R., Wang J., Cong Q., Kinch L.N., Schaeffer R.D. (2021). Accurate prediction of protein structures and interactions using a three-track neural network. Science.

[43] Venkatraman V., Yang Y.D., Sael L., Kihara D. (2009). Protein-protein docking using region-based 3D Zernike descriptors. BMC Bioinform..

[44] Zhou H., Skolnick J. (2011). GOAP: A Generalized Orientation-Dependent, All-Atom Statistical Potential for Protein Structure Prediction. Biophys. J..

[45] Zhou H., Zhou Y. (2009). Distance-scaled, finite ideal-gas reference state improves structure-derived potentials of mean force for structure selection and stability prediction. Protein Sci..

[46] Huang S.-Y., Zou X. (2011). Statistical mechanics-based method to extract atomic distance-dependent potentials from protein structures. Proteins Struct. Funct. Bioinf..

[47] Ng A., Xavier R.J. (2011). Leucine-rich repeat (LRR) proteins: Integrators of pattern recognition and signaling in immunity. Autophagy..

[48] Boden S.A., McIntosh R.A., Uauy C., Krattinger S.G., Dubcovsky J., Rogers W.J., Xia X.C., Badaeva E.D., Bentley A.R., Brown-Guedira G. (2023). Wheat Initiative. Updated guidelines for gene nomenclature in wheat. TAG. Theor. Appl. Genet..

[49] Nagar P., Kumar A., Jain M., Kumari S., Mustafiz A. (2020). Genome-wide analysis and transcript profiling of *PSKR* gene family members in *Oryza sativa*. PLoS ONE.

[50] Zhang Y., Zeng L. (2020). Crosstalk between Ubiquitination and Other Post-translational Protein Modifications in Plant Immunity. Plant Commun..

[51] Khalil H.B., Ehdaeivand M.R., Xu Y., Laroche A., Gulick P.J. (2015). Identification and characterization of rye genes not expressed in allohexaploid triticale. BMC Genom..

[52] Khalil H.B., Brunetti S.C., Pham U.M., Maret D., Laroche A., Gulick P.J. (2014). Characterization of the caleosin gene family in the Triticeae. BMC Genom..

[53] Torii K.U. (2004). Leucine-rich repeat receptor kinases in plants: Structure, function, and signal transduction pathways. Int. Rev. Cytol..

[54] Kamel A.M., Metwally K., Sabry M., Albalawi D.A., Abbas Z.K., Darwish D.B.E., Al-Qahtani S.M., Al-Harbi N.A., Alzuaibr F.M., Khalil H.B. (2023). The Expression of *Triticum aestivum* Cysteine-Rich Receptor-like Protein Kinase Genes during Leaf Rust Fungal Infection. Plants.

