# Supplementary figures and images for: Genome-Wide Characterization and Expression Profiling of Phytosulfokine Receptor Genes (PSKRs) in Triticum aestivum with Docking Simulations of Their Interactions with Phytosulfokine (PSK): A Bioinformatics Study
Source: Genes (Basel). 2024 Oct 9;15(10):1306. doi: 10.3390/genes15101306 (PMC11507999; doi:10.3390/genes15101306)

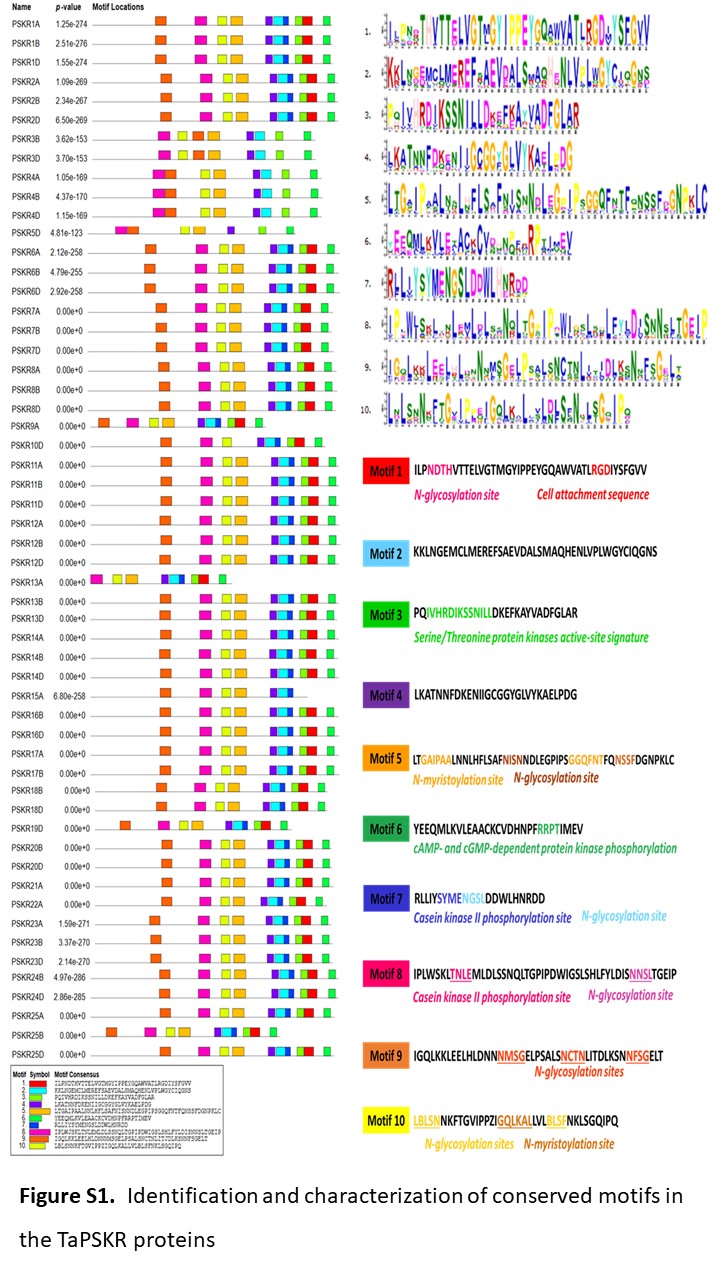

Supplement: Supplementary file 1 [file genes-15-01306-s001.zip › Supplementry Materials/FigureS1.jpg]

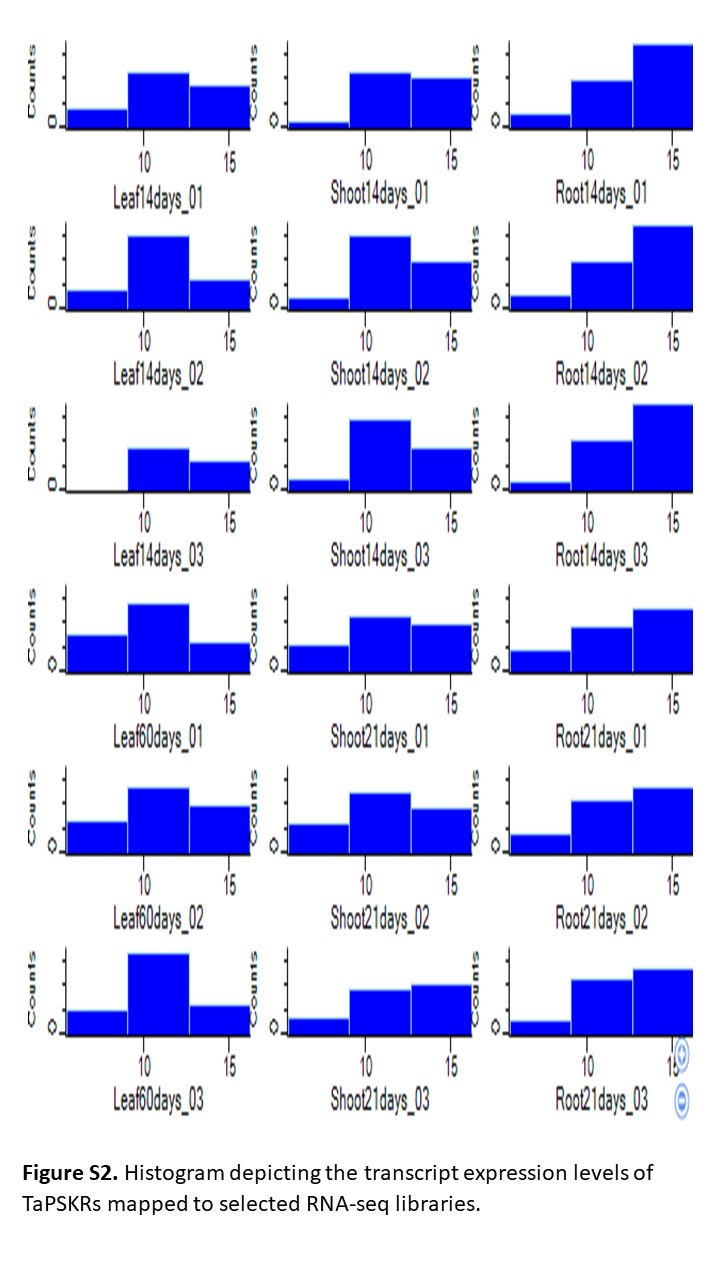

Supplement: Supplementary file 1 [file genes-15-01306-s001.zip › Supplementry Materials/FigureS2.jpg]

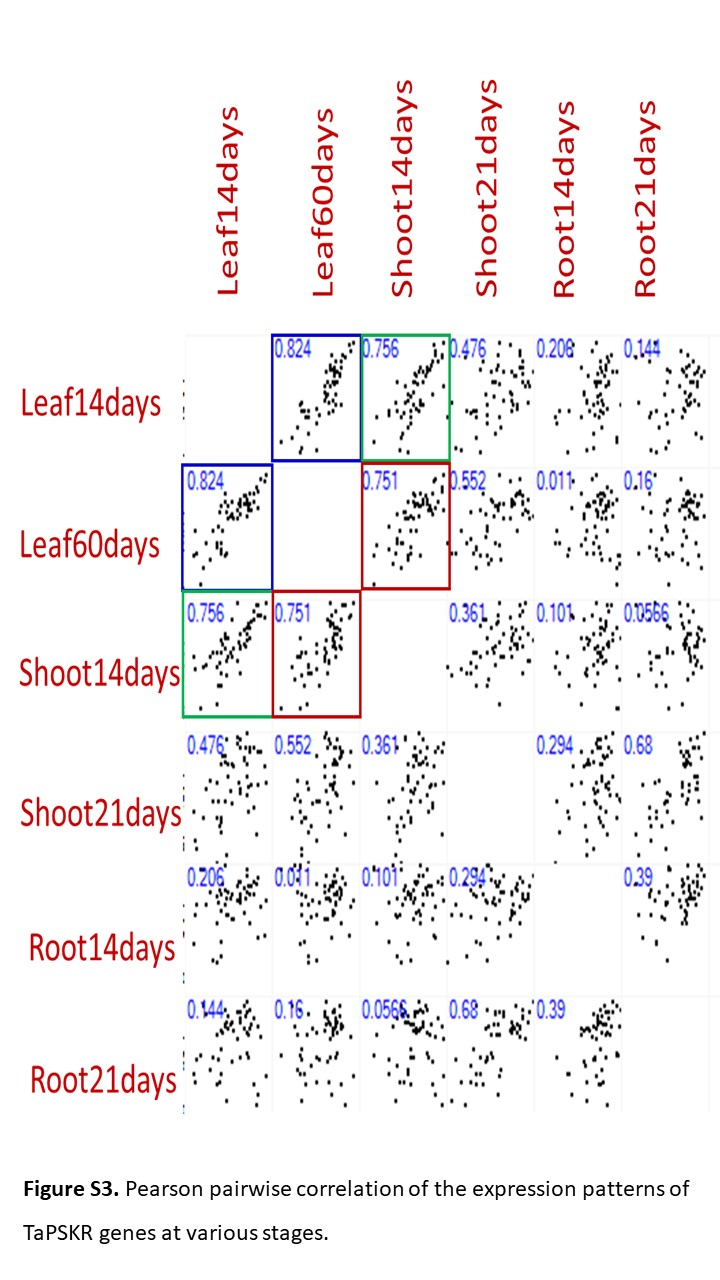

Supplement: Supplementary file 1 [file genes-15-01306-s001.zip › Supplementry Materials/FigureS3.jpg]
